# Cholera in North Kivu: impacts of armed conflict on the resurgence of the epidemic, a narrative review

**DOI:** 10.1097/MS9.0000000000003972

**Published:** 2025-09-30

**Authors:** Joshua Ekouo, Christian Tague, Amos Kipkorir Langat, Aymar Akilimali

**Affiliations:** a Department of Research, Medical Research Circle (MedReC), Goma, DR Congo; b Pan African University for Basic Sciences Technology and Innovation, Nairobi, Kenya

**Keywords:** armed conflict, cholera, humanitarian crisis, North Kivu, waterborne diseases

## Abstract

Cholera remains a major public health threat in the Democratic Republic of Congo, particularly in North Kivu, a province marked by chronic armed conflict and humanitarian crises. This review explores how insecurity, mass displacement, and the collapse of health and sanitation systems have contributed to the resurgence of cholera epidemics. Recurrent armed clashes have not only weakened local health structures but also created living conditions that facilitate the spread of waterborne diseases. Efforts by health authorities and humanitarian organizations have included emergency case management, vaccination campaigns, and water, sanitation, and hygiene interventions, but these have been hampered by persistent insecurity. Lessons learned highlight the need for an integrated, multisectoral response combining emergency measures with long-term investment in health infrastructure, water access, and conflict resolution. Stabilizing the security situation and improving living conditions are essential to sustainably reduce the cholera burden in North Kivu and similar conflict-affected regions.

## Introduction

Cholera is an acute diarrheal infection caused by the ingestion of water or food contaminated by the bacterium *Vibrio cholerae*. This waterborne disease is a major public health problem in many developing countries, particularly in sub-Saharan Africa, where drinking water and sanitation infrastructure remains inadequate^[1]^. According to the World Health Organization (WHO), several million cases are still recorded each year worldwide, with recurring epidemics in regions of high vulnerability^[1,2]^. In the Democratic Republic of Congo (DRC), particularly in the North Kivu province, cholera has been endemic for several decades, with outbreaks exacerbated by repeated security and humanitarian crises. The armed conflicts that have affected this region for several years have caused massive population displacement, the destruction of health infrastructure, and the disruption of health and sanitation services, creating an environment conducive to the spread of waterborne diseases such as cholera. In this context, the upsurge in cholera cases observed in North Kivu during the military clashes illustrates the direct impact of armed conflicts on public health^[3,4]^. This review aims to analyze the impacts of armed conflict on the resurgence of waterborne diseases, particularly cholera, in the context of a humanitarian crisis in North Kivu. It aims to highlight the links between insecurity, population displacement, the failure of health infrastructure, and epidemic outbreaks, while proposing ways to eradicate and prevent future epidemics.

**HIGHLIGHTS**

- Armed conflicts and population displacement in North Kivu have created conditions conducive to the resurgence of cholera.
- Destruction of health infrastructure and limited access to safe water and sanitation have exacerbated the spread of waterborne diseases.
- Children aged under 5 years are particularly affected, with high mortality rates linked to cholera.
- Despite humanitarian interventions (treatment centers, vaccination, and water, sanitation, and hygiene programs), persistent insecurity hampers effective response efforts.
- Sustainable cholera control requires security stabilization, rehabilitation of basic services, and a multisectoral approach.

Recent efforts to standardize AI research reporting, such as the Transparency In The Reporting of Artificial Intelligence (TITAN) guideline, emphasize the critical need for methodological clarity and reproducibility in machine learning studies (TITAN, 2025)^[5]^.

### Epidemiology

Cholera remains a persistent scourge in the DRC, where thousands of cases are recorded each year, making the country one of the most active hotbeds in the world. This waterborne disease remains a major health threat, particularly in the eastern provinces such as North Kivu, which is a region plagued by conflict and mass population displacement, making it one of the epicenters of this epidemic^[6]^.

According to the latest report from the World Health Organization (WHO, 2023), more than 20 000 cases of cholera were confirmed in the DRC in 2023, with a case fatality rate that sometimes exceeds 2%, well above the acceptable threshold. This is due to recurring armed conflicts and large movements of displaced populations which, in a vicious circle, combine instability, poverty, and unsanitary conditions, reinforcing the persistence of the disease. The persistence of armed conflicts in eastern DRC complicates the delivery of care and resources, thus contributing to slowing down the health response^[6,7]^.

Children aged under 5 years, who are particularly vulnerable, pay a heavy price with thousands of preventable deaths each year. Despite vaccination and awareness-raising efforts led by UNICEF and its partners, the resurgence of armed violence in 2024 further complicated access to care, transforming cholera into a “crisis within a crisis”^[7]^. These measures have made it possible, in certain targeted areas, to reduce the number of cases by nearly 30%, illustrating the positive impact of a local and participatory approach^[8,9]^.

The WHO also points out that cholera, although curable, can become fatal if not treated promptly, highlighting the urgent need to improve health coverage and access to safe drinking water, thereby reducing the high prevalence of this disease. The WHO estimates that there are 1.3–4.0 million cases of cholera worldwide each year, and 21 000–143 000 deaths from this disease. The fight against this disease cannot be limited to medical care alone: it also requires an integrated approach, combining public health, development of water and sanitation infrastructure, and even conflict resolution^[2,10]^.

### Armed conflicts and weakened health systems

For more than two decades, North Kivu province has been the scene of recurring armed conflicts involving local and foreign armed groups, with successive waves of violence, attacks on civilians, and mass displacement. These clashes have profoundly affected basic infrastructure, particularly the health system, which was already fragile before the crises^[11]^. Health centers have been looted, destroyed, or deserted, leading to an interruption in the provision of essential health care, including the prevention and management of infectious diseases^[12]^. Conflicts have also led to the forced displacement of thousands of people into makeshift camps that are often overcrowded and poorly equipped, located in areas where access to safe drinking water and sanitation is extremely limited. This context favors the contamination of water by pathogens and the rapid emergence of waterborne diseases, including cholera. Furthermore, insecurity significantly hinders health interventions, whether for epidemiological surveillance, the distribution of medical supplies, or the implementation of vaccination programs. Health workers are sometimes forced to flee or abandon high-risk areas, leaving communities without assistance. Disruptions in the supply chain for medicines, chlorine, vaccines, and WASH kits exacerbate the vulnerability of populations^[1,5,13]^.

Armed conflict in North Kivu has sharply exacerbated cholera outbreaks, with dire consequences for surgical and medical services. Up to 95% of health infrastructure in the worst-affected areas like Kirotshe is inoperable, with 29 out of 31 health centers destroyed or looted. This devastation leaves those needing routine, emergency, and surgical care without functioning facilities or equipment. Hospitals have become overwhelmed, sheltering thousands including displaced children further straining limited resources such as Shortages of Personal Protective Equipment (PPE), pharmaceuticals, and basic kits (dressing and delivery) severely constrain both surgical interventions and the management of infectious cases, including cholera treatment^[14]^. The breakdown of water, sanitation, and hygiene (WASH) facilities leads to inadequate waste management, lack of clean water, and crowded wards, all of which hamper infection control. This increases the risk not only of cholera transmission but also of nosocomial infections affecting all surgical and medical patients^[15]^. Thus, armed conflicts not only disrupt health facilities but also create environmental and social conditions conducive to the resurgence of waterborne diseases. North Kivu, as a region plagued by chronic instability, provides a striking illustration of this link between insecurity and epidemic outbreaks^[16]^.

### Resurgence of cholera and other waterborne diseases

For several years, North Kivu has remained one of the provinces most affected by cholera in the DRC. This waterborne disease, fueled by poor sanitation, experiences recurring outbreaks whose scale is closely linked to the region’s security situation. Recent armed clashes have led to a significant resurgence of cholera cases, particularly in the health zones of Goma, Nyiragongo, Rutshuru, and Masisi. This increase is largely due to the massive displacement of populations, forced to leave their villages and take refuge in makeshift camps, often lacking drinking water, adequate latrines, and basic hygiene facilities^[17,18]^.

Several factors have contributed to the rapid spread of cholera in this degraded humanitarian context. Lack of access to drinking water remains one of the main determinants. In displacement camps and disaster-stricken villages, populations consume surface water or untreated wells, often contaminated by fecal matter. Added to this are very precarious hygiene conditions, marked by the absence of handwashing facilities and under-equipped sanitation infrastructure^[3,19]^. Overcrowding in displacement sites makes contamination control particularly difficult, thus facilitating the fecal-oral transmission of the *V. cholerae bacterium*. Furthermore, poor waste and wastewater management, combined with the stagnation of dirty water in residential areas, constitutes a breeding ground for the spread of the disease^[20]^.

This resurgence of cholera has had considerable health and socioeconomic impacts. From a health perspective, the increase in morbidity and mortality is particularly worrying, especially among children aged under 5 years, who are more vulnerable to severe acute dehydration. Local health facilities, already weakened by the war and lack of resources, quickly found themselves overwhelmed, struggling to manage cases effectively^[9]^. Socially, affected families have suffered economic losses and additional hardships related to the disease, exacerbating their precariousness and further compromising community cohesion and resilience^[21]^.

### Humanitarian responses and control strategies

Faced with the resurgence of cholera in North Kivu in this context of humanitarian crisis, several interventions were implemented by health authorities, nongovernmental organizations (NGOs), and international humanitarian agencies. These actions aimed to contain the epidemic, strengthen case management capacities, and improve the living conditions of displaced and affected populations. However, the implementation of these strategies encountered numerous challenges related to persistent insecurity and logistical constraints^[7,22]^. The first measures consisted of the installation of cholera treatment centers in the most affected areas to ensure rapid and adequate case management. These facilities made it possible to treat patients suffering from severe dehydration and prevent serious complications. At the same time, community awareness campaigns were organized in displacement camps and at-risk neighborhoods to inform the population about the modes of cholera transmission, preventive measures, and the importance of seeking early care. These educational activities, often carried out in collaboration with community leaders and local representatives, played a vital role in reducing the spread of the disease^[23]^. The fight against cholera also relied on WASH interventions. Chlorine tablets, soap, and hygiene kits were distributed in several locations, while boreholes and drinking water points were constructed or rehabilitated to improve access to safe water. Disinfection campaigns for water sources and latrines were also organized, particularly in displacement camps. In addition, the WHO and its partners conducted oral cholera vaccination campaigns in high-risk areas, an effective preventive strategy in precarious humanitarian contexts^[24]^.

Despite these efforts, numerous challenges hampered the optimal implementation of these interventions. Ongoing insecurity in some areas limited access for medical and humanitarian teams, complicating the deployment of response activities and the distribution of resources. The mobility of displaced persons and the instability of camps also hampered the continuity of actions and vaccination coverage^[25]^. Furthermore, weak local health infrastructure and a lack of qualified human resources posed major obstacles to strengthening the health system, which is essential for sustainable epidemic management. This situation underscores the need to integrate emergency responses into a broader strategy to strengthen health systems and improve WASH infrastructure in crisis settings^[26]^.

### Lessons learned and perspectives

The resurgence of cholera in North Kivu, in a context marked by armed conflict and humanitarian crises, highlights deep structural vulnerabilities that must be addressed sustainably. This situation serves as a reminder that public health cannot be separated from the security, economic, and social conditions in which populations live. Epidemics of waterborne diseases, such as cholera, are sensitive indicators of the deterioration of health systems and essential infrastructure, exacerbated by violence and mass displacement^[26,27]^.

Among the key lessons learned, it appears essential to strengthen local capacities in epidemic prevention and management. This involves not only training health personnel and community outreach workers, but also sustainably improving access to drinking water, sanitation, and health infrastructure. Emergency response must be accompanied by structural actions aimed at securing water supplies, developing community latrines, and promoting hygiene in precarious settings, particularly displacement camps and under-equipped peri-urban areas^[28–30]^.

**Figure 1.**
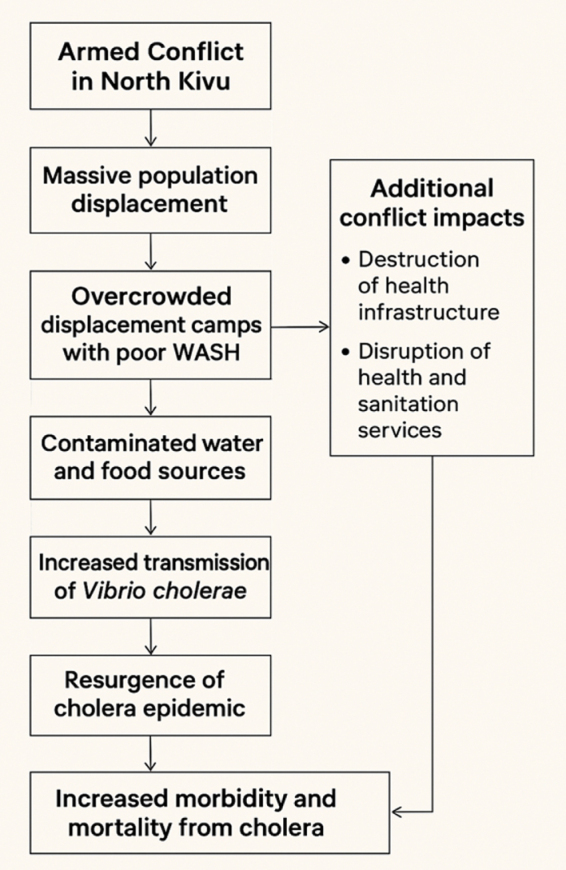
Causal chain between armed conflict and cholera epidemic in North Kivu.

Furthermore, the experience in North Kivu demonstrates the importance of strengthening coordination between humanitarian actors, health authorities, and local communities. Cholera control interventions must be integrated and multisectoral, combining medical care, WASH activities, epidemiological surveillance, and behavior change communication. Oral cholera vaccination has also proven its usefulness in emergency situations, but it must be considered as a complement to traditional prevention and control measures. In the long term, stabilizing the security situation and developing basic infrastructure remain essential conditions for sustainably reducing the epidemic risk and improving health indicators in North Kivu (Fig. 1).

## Conclusion

The health situation in North Kivu alarmingly illustrates the close link between armed conflict, humanitarian crises, and the resurgence of waterborne diseases such as cholera. Recurring clashes have weakened health infrastructure, caused massive population displacement, and created environmental conditions conducive to the spread of epidemics. The resurgence of cholera in this province demonstrates how prolonged security crises undermine public health gains and exacerbate community vulnerability. Despite the efforts of health authorities and humanitarian partners, responses often remain limited by insecurity and logistical constraints. Case management, prevention actions, improvement of WASH conditions, and vaccination campaigns need to be strengthened and integrated into a sustainable, multisectoral approach. To sustainably prevent the resurgence of cholera and other waterborne diseases in this region, it is essential to stabilize the security situation, rehabilitate health infrastructure, and ensure equitable access to safe drinking water and sanitation, particularly for displaced and vulnerable populations. For recommendations directed at the Ministries of Health or NGOs working in North Kivu: prioritize the rapid restoration and reliable supply of WASH services (clean water, latrines, and waste disposal) in all health facilities. Ensure the regular distribution of PPE and sanitation supplies to reduce cross-infection during cholera outbreaks. Finally, this crisis serves as a reminder that in contexts of protracted conflict, public health must remain a top priority and be the subject of strategic investments to preserve the lives and dignity of affected populations.

## Data Availability

Not applicable.
